# Collagen Extraction Optimization from the Skin of the Small-Spotted Catshark (*S. canicula*) by Response Surface Methodology

**DOI:** 10.3390/md17010040

**Published:** 2019-01-09

**Authors:** María Blanco, José Antonio Vázquez, Ricardo I. Pérez-Martín, Carmen G. Sotelo

**Affiliations:** 1 Grupo de Biotecnología y Bioprocesos Marinos, Instituto de Investigaciones Marinas (CSIC), 36208 Vigo, Spain; jvazquez@iim.csic.es (J.A.V.); ricardo@iim.csic.es (R.I.P.-M.); carmen@iim.csic.es (C.G.S.); 2 Laboratorio de Bioquímica de Alimentos, Instituto de Investigaciones Marinas (CSIC), 36208 Vigo, Spain; 3 Laboratorio de Reciclado y Valorización de Materiales Residuales (REVAL), Instituto de Investigaciones Marinas (CSIC), 36208 Vigo, Spain

**Keywords:** fish discards, fish by-products, collagen, cosmetic applications, experimental designs, response surface methodology

## Abstract

The small-spotted catshark is one of the most abundant elasmobranchs in the Northeastern Atlantic Ocean. Although its landings are devoted for human consumption, in general this species has low commercial value with high discard rates, reaching 100% in some European fisheries. The reduction of post-harvest losses (discards and by-products) by promotion of a full use of fishing captures is one of the main goals of EU fishing policies. As marine collagens are increasingly used as alternatives to mammalian collagens for cosmetics, tissue engineering, etc., fish skins represent an excellent and abundant source for obtaining this biomolecule. The aim of this study was to analyze the influence of chemical treatment concentration, temperature and time on the extractability of skin collagen from this species. Two experimental designs, one for each of the main stages of the process, were performed by means of Response Surface Methodology (RSM). The combined effect of NaOH concentration, time and temperature on the amount of collagen recovered in the first stage of the collagen extraction procedure was studied. Then, skins treated under optimal NaOH conditions were subjected to a second experimental design, to study the combined effect of AcOH concentration, time and temperature on the collagen recovery by means of yield, amino acid content and SDS-PAGE characterization. Values of independent variables maximizing collagen recovery were 4 °C, 2 h and 0.1 M NaOH (pre-treatment) and 25 °C, 34 h and 1 M AcOH (collagen extraction).

## 1. Introduction

The small-spotted catshark (*Scyliorhinus canicula*) is one of the most abundant elasmobranchs in the Northeastern Atlantic Ocean [1]. Although its landings are sometimes devoted for human consumption (rendering 10% and 16% of fish weight in the form of skin and viscera by-products respectively), it has low commercial value and very often is captured as by-catch resulting in a very high discard rate reaching 100% in some European fisheries. The reduction of post-harvest fish losses (discards and by-products) by the promotion of a full use of fishing captures is one of the main purposes of EU fishing policies [2]. The full use of fishing captures includes the transformation of raw materials for the isolation/production of molecules that could be used in a wide variety of applications, which is indeed one of the approaches included in the “blue growth” strategy of the European Commission. One interesting bioactive compound which could be obtained from fish discards is collagen. Collagen is the main protein present in animal connective tissue and although there are several types of collagens, type I is the most abundant in the skin and bone of teleost fish [3]. Type I collagen, which is a fibrillar collagen, is a heterotrimeric molecule composed of two α1-chains and one α-2 chain with a similar molecular weight of about 100 kDa [4].

Collagens obtained from marine sources include several *Osteichthyes* and *Chondrichthyes* species, jellyfish, mollusks, sponges and sea cucumbers, among others [5,6,7,8,9,10]. As collagens are being used increasingly as alternatives to mammalian collagens for cosmetics, tissue engineering and other biomedical and pharmaceutical uses, due to safety reasons and ethical or religious constraints, fish skins from discards or by-products represent an excellent and abundant source for obtaining this biomolecule [5,8]. The main difference between marine and mammalian collagen includes a lower content of imino acids (proline and hydroxyproline) in marine collagen, which also influences the lower thermal stability shown by marine collagens [4,6]. In the literature, there is abundant information regarding the extraction of collagen from the skin of different marine species [4,6], however there are only few publications regarding the optimization of the key parameters influencing the process of extraction (temperature, concentration of NaOH and acetic acid and also time of incubation) [11,12,13]. Thus, having in mind the importance of extraction conditions to achieve a higher collagen yield, and although acid-soluble collagen (ASC) has been obtained previously from the skin of the small-spotted catshark [6], the yield was 52%. It is necessary to study the effects of extraction conditions on trying to obtain a higher recovery of collagen from this species.

The collagen extraction process comprises two main steps: the first step consists of the removal of non-collagen proteins and other impurities such as lipids, calcium etc. from the skin, with the aim of increasing the purity of the collagen extracted. To achieve this objective, 0.1 M NaOH is generally used, with different stirring incubation times [14,15,16]. The de-proteinized skin is then washed with cold water until it reaches a neutral pH and filtered. The second step in the extraction process consists of an acidic extraction of the previously NaOH treated skin, commonly using 0.5 M AcOH with different incubation times (48 h, 72 h, etc.) [4,5,17]. All procedures are usually done at 4 °C. After centrifugation, supernatant containing the acid-soluble collagen (ASC) is dialyzed and freeze-dried.

As there are several factors influencing the two-step collagen extraction process (time, temperature, NaOH concentration and AcOH concentration) and there is a need to study the optimal conditions of each variable and also the interactions between them, response surface methodology (RSM) has been employed to predict the optimal experimental conditions. RSM is a tool that has been previously used for the optimization of collagen extraction conditions from the skin of different fish species [12,13,18,19], however none of those studies included all the key optimization parameters influencing the two main steps of the extraction process. Thus, this is the first study optimizing the complete process for the extraction of acid-soluble collagen by means of three variables in each optimization stage: temperature, time and chemical treatment (NaOH or AcOH) concentration, from the skin of the small-spotted catshark.

## 2. Results and Discussion

### 2.1. Alkaline Pre-Treatment of Skin

The average (±standard deviation (SD)) chemical composition of non-treated skin from the small spotted catshark expressed as dry weight is shown in Table 1.

Hydroxyproline (HPro) content was used as an estimation of initial collagen content in the non-treated skins, considering that the ratio of HPro in collagen is 12.5 g of HPro/100 g of collagen [20]. Thus, the determined collagen content was 34.22% (g collagen/100 g dried skin). Collagen recovered (g collagen/100 g of collagen in non-treated skins) was estimated in the solid skin residues and in the filtrated liquid for the 20 experiments carried out during the experimental design, from the Kjeldahl determined nitrogen using a factor of 5.4 [21] (Table S1, Supplementary Material).

Experimental data from Table S1 were modelled using second-order equations (Table 2). These polynomial models describe the correlation between variables and the corresponding response followed the general form defined by Equation (1).

**Figure 1:**
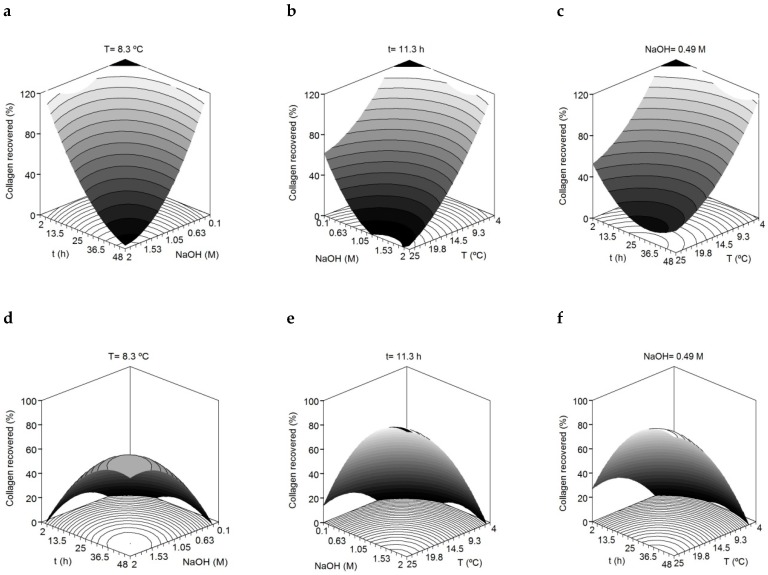
Combined effect of alkali concentration (M), time (t) and temperature (T) on the removal of collagen from the skin of the small-spotted catshark. Collagen recovered in the solid fraction (**a**–**c**). Collagen recovered in the liquid fraction (**d**–**f**).

The R^2^_adj_ values revealed good agreement among experimental and predicted data described by the second-order equations proposed (a high proportion of variability, more than 81% for both solid residue skins and filtrated liquid, was achieved). The consistency of the polynomial equations was validated since the F1 and F2 ratios from F-Fisher test were satisfied in all cases (data not shown). The results of the multivariate analysis showed significant quadratic terms for temperature, NaOH concentration and time (Student’s *t*-test, *p* < 0.05) in the estimated collagen present in both fractions. In the solid fraction, this outcome is graphically translated as a concave surface where the collagen recovery increases with lower temperature, lower concentration of NaOH and low reaction times (Figure 1). The inverse response obtained for temperature, NaOH concentration and time in the filtrated liquid (convex surface) is in agreement with the fact that collagen recovered in the solid fraction is not present in the filtrated liquid fraction. Among the three independent variables, NaOH seems to have a slightly higher effect on collagen recovery in both fractions.

The variables maximizing the recovery of collagen in the solid fraction were 4 °C, 2 h and 0.1 M NaOH. However due to industrial constraints, mainly due to the high cost of low temperature processes, the temperature of 8.3 °C was selected for the next optimization step. Thus, the consensus values for the subsequent acid-soluble collagen extraction step were a temperature of 8.3 °C, a treatment time of 2 h and a NaOH concentration of 0.1 M.

Although previous studies have also shown that a low impact NaOH pre-treatment has a positive effect on the collagen yield, this is the first time that an optimization study has been carried out regarding the skin NaOH pre-treatment. Our results show that as little as two hours of treatment is enough to condition the skin, making it suitable for the posterior acid treatment. Thus, Woo et al. [12] have found that treatment times between 12 h and 36 h and NaOH concentration values between 0.5–1.3 M positively affects the achievement of maximum values of collagen content extracted from yellowfin tuna skin. Zhou and Regenstein [22] found that significant amounts of collagens are lost when pre-treatment conditions include concentration values higher than 0.5 M NaOH, reaction time of 4 days and temperature of 4 °C. Liu et al. [15] have also studied the effect of different alkaline pre-treatment conditions on the acid-soluble collagen obtained from grass carp, concluding that temperature ranges of 4–20 °C for pre-treatment conditions and NaOH concentration between 0.05 and 0.1 M were adequate. Wang et al. [13] also employed 0.1 M NaOH to remove non-collagenous proteins from the skin of grass carp with low temperature (4 °C) but higher reaction time (6 h). These results suggest that the efficiency of alkaline pre-treatment may vary between fish species and also between temperature, time and NaOH concentration conditions, highlighting the importance of specific two-step optimization studies for different species including these three variables.

### 2.2. Acid-Soluble Collagen (ASC) Extraction Stage

**Figure 2:**
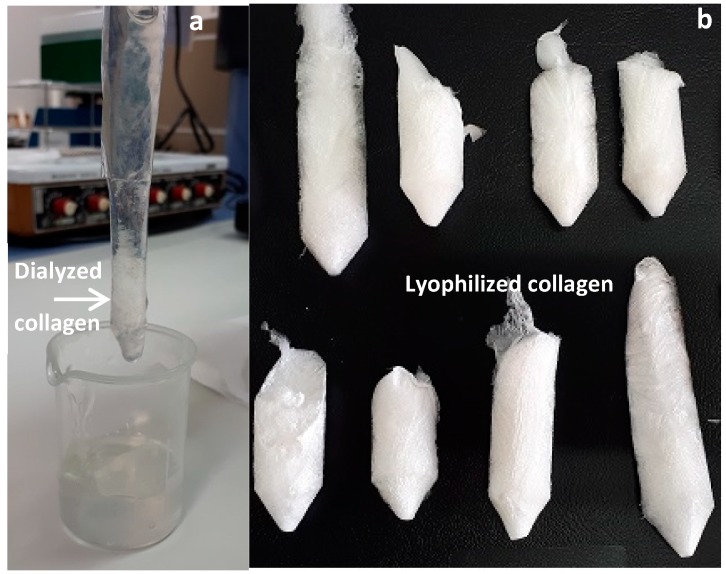
Dialyzed (**a**) and lyophilized (**b**) collagens obtained in each of the 20 experiments developed for the acid-soluble collagen extraction stage experimental design.

The next experiment was designed for the optimization of collagen extraction in acidic media using NaOH pre-treated skin. In this case, the combined effect of acetic acid concentration, temperature and time of processing on collagen production was studied. The average (±SD) chemical composition of NaOH treated skins (under the optimal consensus values obtained in the first experimental optimization stage, expressed as percentage of dry weight) used for this second experimental design was 76.55 ± 1.22% of moisture content; 56.71 ± 0.61% of protein content; 0.59 ± 0.10% of lipid content and 46.49 ± 0.38% of ash content. Compared to the approximate composition of non-treated skins, the protein and lipid content decreased significantly (Kruskal–Wallis test for protein: chi-square = 5.398, d.f. = 1, *p* = 0.020; ANOVA for lipid: F_1,4_ = 299.483, *p* < 0.01), confirming the removal of unwanted materials [23]. The significantly higher ash content observed in NaOH treated skin (ANOVA for ash: F_1,4_ = 1758.801, *p* < 0.01 is due to the NaOH added. A representation of the lyophilized collagen obtained in some of the 20 experiments is shown in Figure 2. The corresponding amino acid composition from all collagens is summarized in Table 3. In addition, the yields of lyophilized collagen recovered varied between 18.33% and 49.65% and are defined in Table S2.

**Figure 3:**
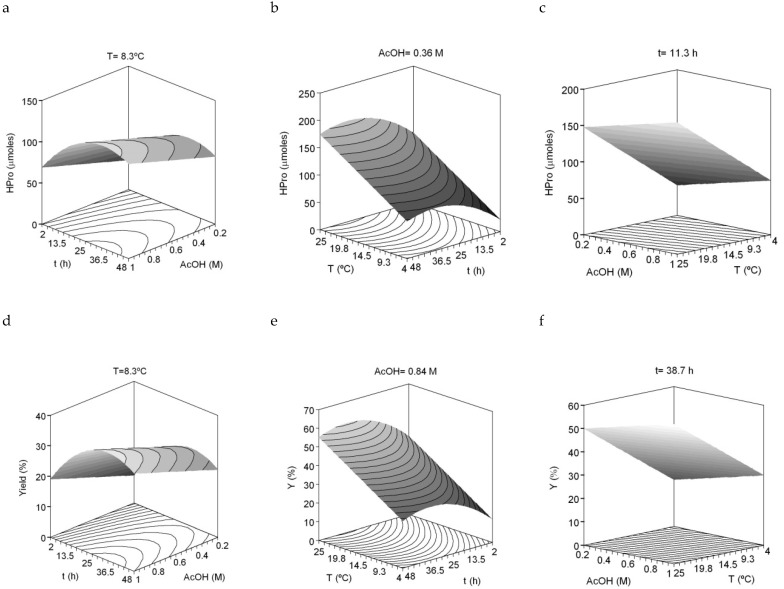
Combined effect of acetic acid (AcOH), time (t) and temperature (°C) on HPro released (**a**–**c**) and collagen yield (**d**–**f**) produced from *S. canicula* skins.

The dependent variables (responses) evaluated were HPro, Gly, Pro and the sum of Pro + HPro (imino acids) as well as the yield of collagen recovered. Table 4 summarizes the equations obtained from the mathematical modelling and multivariable statistical analysis of the experimental responses mentioned. The accuracy between experimental and theoretical data were remarkable with values of R^2^_adj_ > 0.85. The robutness of the different response selected and the reproducibility of collagen production was confirmed by the fact that equations and theoretical three-dimensional (3D) surfaces were similar in all cases studied (Figure 3). As in the previous factorial design, the consistency of the equations was also found: all ratios F1–F4 were validated (data not shown). Finally, the values of the independent variables which maximize the recovery of collagen were a temperature of 25 °C, a time of 34 h and a concentration of 1 M acetic acid. Using these optimal extraction conditions, the yield of collagen obtained was 61.24% (g of collagen/100 g of initial collagen in skin), which is higher than that obtained previously [6].

Previously published results on acid-soluble collagen extraction from the skin of the small-spotted catshark [6] showed lower yield values as the extraction conditions were different than the optimum values presented here. Several studies have also focused on the extraction and characterization of acid-soluble collagen from different marine fish species, traditionally using 0.5 M acetic acid at around 4 °C [22] without a previous optimization study [24,25]. In recent decades, several manuscripts have addressed the study of the optimization conditions of collagen extraction from different sources; however, not many of those optimize the complete extraction procedure, including both the alkaline and the acidic stages. Thus, Wang et al. [13] found higher yields of acid-soluble collagen from the skin of grass carp with increased acetic acid concentration (up to 0.5 M) and increased reaction times (up to 32 h), while the optimum temperature differs with different levels of acetic acid or reaction time. The collagen yield reported by these authors was lower than the one obtained in this study. As in the previous alkaline pre-treatment optimization stage, the efficiency of the acidic extraction stage varies between fish species and also with temperature, time and concentration of acetic acid, suggesting the great importance of specific optimization studies for different species including the three variables involved in the process.

**Figure 4:**
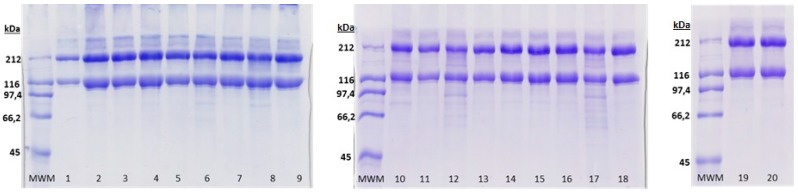
SDS-PAGE (7%) showing acid-soluble collagen (ASC) obtained in each of the 20 experiments developed for the acid-soluble collagen extraction experimental design. MWM: molecular weight marker.

As shown in Figure 4, ASC extracted under the different experimental conditions used in this work resulted in similar electrophoretic patterns, which consisted of the typical heterotrimer collagen structure containing two identical α_1_ chains (approximately 120 kDa) and one α_2_ chain (approximately 110 kDa) in the molecular form of [α_1_(I)]_2_ α_2_(I)], and one β dimer of about 200 kDa [6]. The band intensity of the α_1_ chain was not two-fold higher than that of α_2_ chain; in fact, the α_2_ chain is hardly visible. This fact, together with the high intensity of the β dimer, might suggest the existence of higher crosslink degree between α_2_ chain in elasmobranchs This has also been found for other elasmobranchs where the α_2_ chains are scarcely visible while the β dimer bands are stronger than in other teleosts [17,26,27]. A γ-component can be also seen in all ASC obtained, similarly to previously reported results by Sotelo et al. [6]. The collagen obtained in some of the experimental conditions (corresponding to Experiments 6, 8, 10 and 12) present a few bands below 100 kDa. These low molecular weight components might be the result of the particular extraction conditions on which those collagens were obtained: the highest temperatures, times and AcOH concentrations or the combination of them (further research on the characterization of those components using ionic exchange chromatography might be interesting, however it exceeds the objectives of this study).

## 3. Material and Methods

### 3.1. Biological Samples and Compositional Analysis

**Figure 5:**
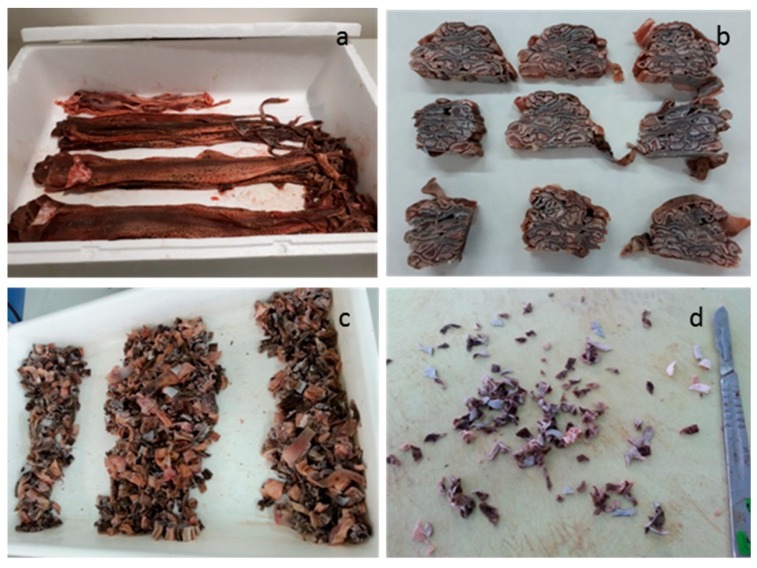
Small spotted catshark skin sampling: Skins stretched and aligned on top of each other (**a**); selected central portions of skins (**b**); homogeneized cutsobtained from the selected central parts of skins using scissors and divided in three for sampling purposes (**c**); small pieces finally obtained using a scalper (**d**).

Small-spotted catshark (*Scyliorhinus canicula*) individuals obtained approximately 12 h after capture from a local market in Vigo (Northwestern Spain) were manually skinned (ES360570202001 by Galicia Government). Skins were stretched and aligned on top of each other (Figure 5a) in order to select only the central part of the skins (Figure 5b) with the aim of obtaining a homogeneous material. The selected central parts were mechanically cut into small pieces (5 × 5 cm^2^) (Figure 5c) and then each of those pieces were manually cut into smaller pieces (0.5 × 0.5 cm^2^) (Figure 5d), mixed thoroughly, separated in sealed plastic bags each containing 5 g of skin and stored at −20 °C until used for the experimental designs.

The chemical composition of the skin was evaluated in triplicate by analyzing crude protein, ash, moisture and fat content. Total nitrogen was determined with the Kjeldahl method [28] in a DigiPREP HT digestor (SCP Science, Baie-d’Urfe, QC, Canada), DigiPREP 500 fully automatic steam distillation (SCP Science, Baie-d’Urfe, QC Canada) and a TitroLine easy titration unit (Schoot, Mainz, Germany), and crude protein content was calculated as total nitrogen multiplied by 6.25. Fat content was determined by the method of Bligh and Dyer [29]. Moisture was determined after heating the sample at 105 °C for 24 h, and ash content was determined after heating the sample for 24 h at 550 °C [28].

The hydroxyproline content in the skin was determined according to the procedure described in Blanco et al. [30] and used for the estimation of initial collagen content in the untreated skin, considering that the ratio of HPro in collagen is 12.5 g of HPro/100 g of collagen [20].

### 3.2. Experimental Design and Statistical Analysis

In this work, two experimental designs were performed to analyze the influence of chemical treatment concentration, temperature and time on the extractability of collagen from the skin of the small-spotted catshark. First, the effect of temperature (T), concentration of NaOH (M) and time (t) on the efficiency of removing non-collagenous proteins was studied (alkaline pre-treatment). Then, the effect of temperature (T), concentration of acetic acid (M) and time (t) on the efficiency of extracting acid-soluble collagen (ASC) was optimized (acid-soluble collagen extraction stage). In both cases, the factorial experiments were rotatable second-order designs with six replicates in the center of the experimental domains [31].

#### 3.2.1. Alkaline Pre-Treatment Experimental Design

The conditions of the independent variables studied in the pre-treatment experimental design were: temperature in the range of 4–25 °C, concentration of NaOH in the range of 0.1–2 M and intervals of time between 2–48 h (Table 5). The values of independent variables were selected from previously reported studies to cover a wide range of conditions in order to obtain the values that maximize the isolation of collagen and to reduce the times and concentrations needed for the bioproduction of *S. canicula* collagen. The most common times and NaOH concentration values used in the literature ranged from 1–36 h and 0.05–1.3 M NaOH. The temperature preferentially chosen in the literature for removing other non-collagen proteins ranged from 4–20 °C [11,12,13].

The conditions which were maintained as constants were the solid (skin):alkaline solution ratio of 1:10 and high agitation (200 rpm). The reactions were developed in a stirred and thermostated reactor (100 mL). After each of the 20 alkali treatments, the solutions were filtered using a 35 µm membrane. The filtrate was measured, centrifuged and the supernatant collected to be analyzed in terms of collagen content which was determined by means of total nitrogen content according to the Kjeldahl method [28]. The skin residue of the filter was weighed and also analyzed in terms of total nitrogen content. The dependent variable studied was collagen/initial collagen in skin rate, for both the collagen recovered in the skin residues and the collagen measured in the filtered solution.

#### 3.2.2. Acid-Soluble Collagen Extraction Stage Experimental Design

**Figure 6:**
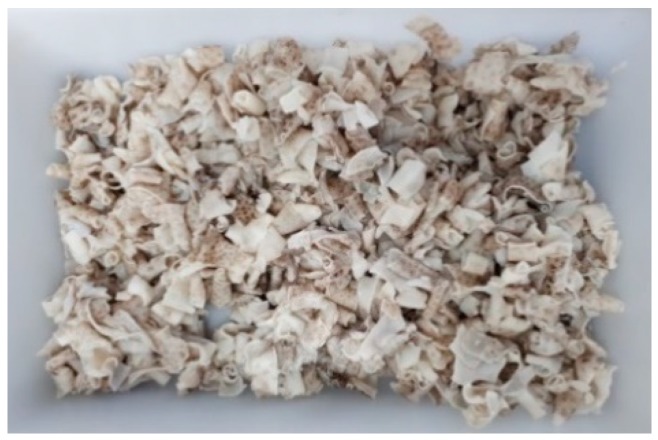
Filtered and washed NaOH treated skins used for the acid-soluble collagen extraction stage of the experimental design.

Skins (500 g) obtained as explained in Section 2.1 were introduced in a stirred and thermostated 5 L reactor connected to a pH electrode and a temperature probe (Afora S.A., Barcelona, Spain). Based on the consensus values obtained in the alkaline pre-treatment experimental design, skins were treated with NaOH and then filtered using a 200 µm membrane. The liquid was removed, and the skins were washed with distilled water until neutral pH was achieved (Figure 6), weighed and divided into 5 g sealed plastic bags which were frozen at −20 °C until used for the experimental design. NaOH pre-treated skins (10 g) were used for approximate compositional analysis. Differences in approximate composition between non-NaOH treated skins and NaOH treated skins were statistically analyzed. Prior to analysis, data were checked for normality and homoscedasticity using the Kolmogorov–Smirnov and Levene tests, respectively. The Kolmogorov–Smirnov test showed that protein data and their transformations did not fit with the assumptions of normality. As a consequence, non-parametric statistics were used for these data. Differences in lipids and ashes were compared by one-way ANOVA, with NaOH treated or non-treated skins as the between-subject effect. The Kruskal–Wallis test, the non-parametric equivalent of a one-way ANOVA, was used to examine variations between treated and non-treated skins in protein content. Significance levels were set at *p* < 0.05. Statistical tests were performed with IBM SPSS Statistics 23. 0 (IBM Corp., Armonk, NY, USA).

The conditions of the independent variables studied in the collagen extraction stage of the experimental design were: temperature in the range of 4–25 °C, concentration of acetic acid (AcOH) in the range of 0.2–1 M and intervals of time between 2–48 h (Table 4). The conditions that were maintained as constants were solid (skin residue): alkaline solution ratio of 1:10 and high agitation (200 rpm). The values of independent variables were again selected from previously reported studies to cover a wide range of conditions in order to obtain the values that maximize the isolation of collagen and to reduce the time and concentration needed for the bioproduction of *S. canicula* collagen. The most common times and acetic acid concentration values used in the literature ranged from 1–16 h and 0.1–1 M acetic acid. The temperature preferentially chosen in the literature for the acid extraction of collagen ranged from 4–30 °C [12,13,24].

The reactions were developed in the same reactor as the pre-treatment experimental design. After each one of the 20 acid experiments, the solutions were filtered using a 200 µm membrane. The filtrated solution was collected, measured, dialyzed and freeze-dried. The freeze-dried collagen was weighed and characterized in terms of collagen content (determined by means of hydroxyproline (HPro), proline (Pro) and glycine (Gly) content), yield of collagen recovered and SDS-PAGE characterization (the content of other amino acid was also analyzed). The dependent variables studied were yield of collagen (as a percentage of collagen recovered/initial collagen in skin) and contents of the collagen characteristic amino acids: HPro, Pro and Gly.

#### 3.2.3. Mathematical Modelling and Statistical Analysis

The experimental results of the factorial designs were modelled by second-order polynomial equations as [31]:

(1) $$Y=b_{0}+\overset{n}{\underset{i=1}{\sum }}b_{i}X_{i}+\underset{j>i}{\overset{n-1}{\underset{i=1}{\sum }}}\overset{n}{\underset{j=2}{\sum }}b_{ij}X_{i}X_{j}+\overset{n}{\underset{i=1}{\sum }}b_{ii}X_{i}^{2}$$

where *Y* represents the response to be modelled; *b*_0_ is a constant coefficient, *b_i_* is the coefficient of linear effect, *b_ij_* is the coefficient of interaction effect, *b_ii_* is the coefficient of squared effect, *n* is the number of variables and *X_i_* and *X_j_* define the independent variables. The statistical significance of the coefficients was verified by means of Student’s *t*-test (α = 0.05), goodness-of-fit was established as the adjusted determination coefficient (R^2^_adj_) and the model consistency was determined by the Fisher F test (α = 0.05) using the following mean squares ratios (Table 6):

### 3.3. Amino Acid Characterization of Acid-Soluble Collagen

Amino acids were determined in the freeze-dried collagen obtained in each one of the 20 experimental points. For this purpose, acid hydrolysis was conducted with 6 N hydrochloric acid containing 0.1% phenol under an inert atmosphere by heating to 110 °C for 24 h. Then, HCl was removed from the hydrolysate by vacuum. The hydrolysate was resuspended in 20–50 µL of 0.2 M sodium citrate buffer (pH 2.2), to which a known amount of norleucine was added as an internal standard and applied to an automated amino acid analyzer (Biochrom30 Amino Acid Analyzer, Biochrom, UK).

### 3.4. SDS-PAGE Characterization of Acid-Soluble Collagen

Samples (1 mg/mL) were prepared in sample buffer containing 10.52% glycerol, 21% Sodium Dodecyl Sulfate (SDS) (10%), 0.63% Dithiothreitol (DTT)and 0.5 M Tris HCl (pH 6.8) and heated for 5 min at 100 °C. An aliquot (8 µL) of this mixture was applied to each well in 7% polyacrylamide separating gels. Gels (100 mm × 750 mm × 0.75 mm) were prepared according to the procedure of Laemmli [32] and were subjected to electrophoresis at 20 mA using a Mini-Protean II Cell (Bio-Rad, Hercules, CA, USA). Following electrophoresis, the gels were stained with 0.04% Coomassie Blue in 25% *v*/*v* ethanol and 8% *v*/*v* acetic acid for 2 h. Excess stain was removed with several washes of destaining solvent (25% *v*/*v* ethanol, 8% *v*/*v* acetic acid). Molecular weights of subunits of ASC (acid-soluble collagen) were estimated using molecular weight standards from BIO-RAD (Hercules, CA, USA) SDS-PAGE standards high range: myosin (200 kDa); β-galactosidase (116 kDa); phosphorylase B (97 kDa); bovine serum albumin (66 kDa); ovalbumin (45 kDa).

## 4. Conclusions

This is the first study optimizing the complete process for the extraction of acid-soluble collagen by means of three variables (temperature, time and chemical treatment concentration) from the skin of the small-spotted catshark using response surface methodology. Two-stage optimizations (alkali pre-treatment and acid extraction) of the collagen extraction process should be accomplished in a species-specific approach due to the variability of collagen extracted from different species (regarding its structure and chemical differences (for example, variations in the amino acid composition)). The variables maximizing the recovery of collagen in the first stage of extraction (alkaline pre-treatment) were 4 °C, 2 h and 0.1 M NaOH. The variables maximizing the recovery of collagen in the second stage of extraction (acid-soluble collagen extraction stage) were 25 °C, 34 h and 1 M AcOH with a yield of 61.24%. The results obtained in this study might be helpful for a potential collagen extraction upscaling study.

## Figures and Tables

**Table 1:** Approximate composition (media ± standard deviation (SD)) expressed as percentage of dry weight of non-treated skin from the small-spotted catshark.

	Composition (%)			
	Moisture	Protein	Lipid	Ash
Non-treated skin	62.22 ± 0.48	69.24 ± 0.67	2.72 ± 0.18	35.13 ± 0.26

**Table 2:** Second-order equations describing the effect of temperature (T), time (t) and concentration of NaOH (M) on the efficiency of collagen recovery (%) from the skin of the small-spotted catshark. The coefficient of adjusted determination (R^2^_adj_) is also shown. Optimum (opt) values of each independent variable to obtain maximum responses are also shown.

	Polynomial Equations	R^2^_adj_	T_opt_ (°C)	t_opt_ (h)	NaOH_opt_ (M)
Liquid	Collagen (%) = 87.9 + 26.2 × T + 14.4 × NaOH + 12.7 × t − 5.9 × T × NaOH × t − 7.9 − T^2^ − 9.6 × NaOH^2^ − 9.1 × t^2^	0.846	25	48	2
Solid	Collagen (%) = 14.4 − 26.5 × T − 17.8 × NaOH − 14.9 × t + 3.7 × T × NaOH + 7.04 × T × t − 4.5 × NaOH × t + 6.6 × T × NaOH × t + 6.9 × T^2^ + 11.7 × NaOH^2^ + 9.3 × t^2^	0.811	4	2	0.1

**Table 3:** Hydroxyproline (HPro), Proline (Pro) and Glycine (Gly) content in lyophilized extracted collagen obtained in each of the 20 experiments developed for the acid-soluble collagen extraction stage of the experimental design. Real values of independent variables are indicated, as well as the codified values (in brackets).

N° Experiment	T (°C)	Acetic Acid (M)	t (h)	Micromole in Lyophilized Extracted Collagen		
				OHPro	Pro	Gly
1	8.26 ((−1))	0.36 (−1)	11.33 (−1)	65.12	102.24	367.69
2	20.74 ((1))	0.36 (−1)	11.33 (−1)	151.34	237.70	854.61
3	8.26 ((−1))	0.84 (1)	11.33 (−1)	87.58	137.44	494.40
4	20.74((1))	0.84 (1)	11.33 (−1)	144.44	226.88	815.48
5	8.26 ((−1))	0.36 (−1)	38.67 (1)	81.01	127.18	456.98
6	20.74 ((1))	0.36 (−1)	38.67 (1)	168.79	265.12	952.75
7	8.26 ((−1))	0.84 (1)	38.67 (1)	121.52	191.05	686.78
8	20.74 ((1))	0.84 (1)	38.67 (1)	174.42	274.06	985.54
9	4.00 (−1.682)	0.60 (0)	25.00 (0)	68.59	107.72	387.32
10	25.00 (1.682)	0.60 (0)	25.00 (0)	168.85	265.32	953.85
11	14.50 (0)	0.20 (−1.682)	25.00 (0)	108.93	171.04	614.92
12	14.50 (0)	1.00 (1.682)	25.00 (0)	155.82	244.70	879.74
13	14.50 (0)	0.60 (0)	2.00 (−1.682)	71.32	112.12	403.03
14	14.50 (0)	0.60 (0)	48.00 (1.682)	131.02	205.82	740.10
15	14.50 (0)	0.60 (0)	25.00 (0)	116.16	182.34	655.82
16	14.50 (0)	0.60 (0)	25.00 (0)	131.89	207.09	744.66
17	14.50 (0)	0.60 (0)	25.00 (0)	139.18	218.56	785.62
18	14.50 (0)	0.60 (0)	25.00 (0)	158.14	248.32	892.94
19	14.50 (0)	0.60 (0)	25.00 (0)	141.82	221.77	797.30
20	14.50 (0)	0.60 (0)	25.00 (0)	134.02	210.51	756.67

**Table 4:** Second-order equations describing the effect of temperature (T), time (t) and concentration of AcOH (M) on the collagen recovery by means of HPro, Gly, Pro, HPro + Pro and yield determination, from the skin of the small-spotted catshark. The coefficient of adjusted determination (R^2^_adj_) is also shown. Optimum values of each independent variable to obtain maximum responses are also shown.

Polynomial Equations	R^2^_adj_	T_opt_ (°C)	t_opt_ (h)	AcOH_opt_ (M)
Pro (µmoles) = 214.4 + 52.1 × T + 16.2 × AcOH + 22.8 × t − 17.1 × t^2^	0.860	25	34.2	1
HPro (µmoles) = 136.5 + 33.1 × T + 10.3 × AcOH + 14.5 × t − 10.9 × t^2^	0.860	25	34.2	1
Gly (µmoles) = 770.7 + 187.1 × T + 58.3 × AcOH + 81.8 × t − 61.4 × t^2^	0.860	25	34.2	1
HPro + Pro (µmoles) = 350.9 + 85.2 × T + 26.5 × AcOH + 37.2 × t − 28.0 × t^2^	0.860	25	34.2	1
Yield (%) = 39.2 + 9.3 × T + 3.1 × AcOH + 4.1 × t − 3.4 × t^2^	0.853	25	34.2	1

**Table 5:** Experimental domain and codification of independent variables in the second-order rotatable designs developed for collagen extraction from *S. canicula* skin.

	Alkaline Pre-Treatment			Acid Extraction		
Coded Values	T (°C)	NaOH (M)	t (h)	T (°C)	AcOH (M)	t (h)
−1.68	4.0	0.10	2.0	4.0	0.20	2.0
−1	8.3	0.49	11.3	8.3	0.36	11.3
0	14.5	1.05	25.0	14.5	0.60	25.0
+1	20.7	1.61	38.7	20.7	0.84	38.7
+1.68	25.0	2.00	48.0	25.0	1.00	48.0
Codification: V_c_ = (V_n_ − V_0_)/ΔV_n_ Decodification: V_n_ = V_0_ + (ΔV_n_ × V_c_) V_n_ = natural value of the variable to codify ΔV_n_ = increment of V_n_ for unit of V_c_ V_0_ = natural value in the centre of the domain V_c_ = codified value of the variable						

**Table 6:** Fisher F tests used to check the consistency of polynomial equations.

	The Model is Acceptable When:
F1 = Model/Total error	F1 ≥ $F_{den}^{num}$
F2 = (Model + Lack of fitting)/Model	F2 ≤ $F_{den}^{num}$
F3 = Total error/Experimental error	F3 ≤ $F_{den}^{num}$
F4 = Lack of fitting/Experimental error	F4 ≤ $F_{den}^{num}$

$F_{den}^{num}$ are the theoretical values to α = 0.05 with the corresponding degrees of freedom for numerator (num) and denominator (den). All fitting procedures, coefficient estimates and statistical calculations were performed on a Microsoft Excel spreadsheet.

## Supplementary Materials

The following are available online at http://www.mdpi.com/1660-3397/17/1/40/s1, Table S1: Experimental domains and codification of independent variables in the factorial rotable design executed to study the optimal conditions for removing proteins different of collagen from the skin of small-spotted catshark, Table S2: Experimental domains and codification of independent variables in the factorial rotable design executed to study the optimal conditions for extraction of acid soluble collagen from the skin of small-spotted catshark.
