# Supplementary material for: Collagen Extraction Optimization from the Skin of the Small-Spotted Catshark (S. canicula) by Response Surface Methodology
Source: Mar Drugs. 2019 Jan 9;17(1):40. doi: 10.3390/md17010040 (PMC6356800; doi:10.3390/md17010040)
Supplement: Supplementary file 1 [file marinedrugs-17-00040-s001.pdf]

Supplementary material

Table S1. *Experimental domains and codification of independent variables in the factorial rotable design executed to study the optimal conditions for removing proteins different of collagen from the skin of small-spotted catshark. Recovered collagen (g collagen/ 100 g initial collagen in skin) in both skin residues and filtrated liquid obtained after filtration is also presented.*

Experiment number	T (ºC)	NaOH (M)	t (h)	T cod	NaOH cod	t cod	Recovered collagen (g) in skin residue/100 g initial collagen	Recovered collagen (g) in filtrated liquid/100 g initial collagen
1	8.26	0.49	11.33	-1.000	-1.000	-1.000	99.70	16,61
2	20.74	0.49	11.33	1.000	-1.000	-1.000	24,38	78,21
3	8.26	1.61	11.33	-1.000	1.000	-1.000	92.05	19,29
4	20.74	1.61	11.33	1.000	1.000	-1.000	5,25	95,19
5	8.26	0.49	38.67	-1.000	-1.000	1.000	84.38	20,81
6	20.74	0.49	38.67	1.000	-1.000	1.000	10,93	92,68
7	8.26	1.61	38.67	-1.000	1.000	1.000	32,34	61,38
8	20.74	1.61	38.67	1.000	1.000	1.000	0,00	100
9	4.00	1.05	25.00	-1.682	0.000	0.000	59,75	33,84
10	25.00	1.05	25.00	1.682	0.000	0.000	4,24	99.34
11	14.50	0.10	25.00	0.000	-1.682	0.000	91,41	23,63
12	14.50	2.00	25.00	0.000	1.682	0.000	0,00	100
13	14.50	1.05	2.00	0.000	0.000	-1.682	71,17	31,18
14	14.50	1.05	48.00	0.000	0.000	1.682	6,24	95,19
15	14.50	1.05	25.00	0.000	0.000	0.000	13,73	87,00
16	14.50	1.05	25.00	0.000	0.000	0.000	16,58	96,25
17	14.50	1.05	25.00	0.000	0.000	0.000	14,12	84,81
18	14.50	1.05	25.00	0.000	0.000	0.000	17,28	82,81
19	14.50	1.05	25.00	0.000	0.000	0.000	8,05	92,07
20	14.50	1.05	25.00	0.000	0.000	0.000	17,30	84,10

Table S2. *Experimental domains and codification of independent variables in the factorial rotable design executed to study the optimal conditions for extraction of acid soluble collagen from the skin of small-spotted catshark. Collagen yields (g lyophilized collagen obtained in each experimental conditions/100 g of initial collagen in skin) are also presented.*

Experiment number	T (ºC)	Acetic acid (M)	t (h)	T cod	Acetic cod	t cod	Collagen yield (%)
1	8.26	0.36	11.33	-1.000	-1.000	-1.000	18.33
2	20.74	0.36	11.33	1.000	-1.000	-1.000	41.45
3	8.26	0.84	11.33	-1.000	1.000	-1.000	25.17
4	20.74	0.84	11.33	1.000	1.000	-1.000	40.49
5	8.26	0.36	38.67	-1.000	-1.000	1.000	22.43
6	20.74	0.36	38.67	1.000	-1.000	1.000	46.64
7	8.26	0.84	38.67	-1.000	1.000	1.000	33.78
8	20.74	0.84	38.67	1.000	1.000	1.000	49.65
9	4.00	0.60	25.00	-1.682	0.000	0.000	19.28
10	25.00	0.60	25.00	1.682	0.000	0.000	48.42
11	14.50	0.20	25.00	0.000	-1.682	0.000	30.50
12	14.50	1.00	25.00	0.000	1.682	0.000	43.50
13	14.50	0.60	2.00	0.000	0.000	-1.682	19.97
14	14.50	0.60	48.00	0.000	0.000	1.682	37.07
15	14.50	0.60	25.00	0.000	0.000	0.000	33.10
16	14.50	0.60	25.00	0.000	0.000	0.000	36.93
17	14.50	0.60	25.00	0.000	0.000	0.000	40.90
18	14.50	0.60	25.00	0.000	0.000	0.000	45.69
19	14.50	0.60	25.00	0.000	0.000	0.000	39.94
20	14.50	0.60	25.00	0.000	0.000	0.000	39.26
